# 2-Amino-1-(3-sulfonato­prop­yl)pyridinium monohydrate

**DOI:** 10.1107/S1600536811004107

**Published:** 2011-02-05

**Authors:** Farook Adam, Tammar H. Ali, Chien-Wen Kueh, Mohd Mustaqim Rosli, Hoong-Kun Fun

**Affiliations:** a School of Chemical Sciences, Universiti Sains Malaysia, 11800 USM, Penang, Malaysia; b X-ray Crystallography Unit, School of Physics, Universiti Sains Malaysia, 11800 USM, Penang, Malaysia

## Abstract

In the title compound, C_8_H_12_N_2_O_3_S·H_2_O, inter­molecular O—H⋯O and N—H⋯O hydrogen bonds and weak C—H⋯O inter­actions, which form *R*_1_^2^(6) and *R*_2_^2^(12) ring motifs, link the components into a three-dimensional network.

## Related literature

For applications of sulfopropyl derivatives, see: Adamczyk & Rege (1998 ▶). For the biological activity of 2-amino­pyridine, see: Salimon *et al.* (2009 ▶). For a related structure, see: Koclega *et al.* (2007 ▶). For the title compound as a heterogeneous catalyst, see: Jayamurugan *et al.* (2009 ▶). For hydrogen-bond motifs, see: Bernstein *et al.* (1995 ▶). For the stability of the temperature controller used in the data collection, see: Cosier & Glazer (1986 ▶).
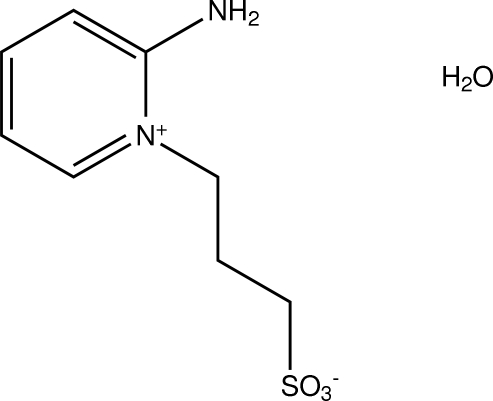


## Experimental

### 

#### Crystal data

C_8_H_12_N_2_O_3_S·H_2_O
*M* *_r_* = 234.27
Monoclinic, 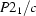

*a* = 9.0771 (3) Å
*b* = 16.6307 (7) Å
*c* = 7.4393 (3) Å
β = 112.794 (1)°
*V* = 1035.32 (7) Å^3^
*Z* = 4
Mo *K*α radiation
μ = 0.31 mm^−1^
*T* = 100 K
0.51 × 0.14 × 0.14 mm

#### Data collection

Bruker SMART APEXII CCD area-detector diffractometer
Absorption correction: multi-scan (*SADABS*; Bruker, 2009 ▶) *T*_min_ = 0.858, *T*_max_ = 0.958
15075 measured reflections
4050 independent reflections
3694 reflections with *I* > 2σ(*I*)
*R*_int_ = 0.023

#### Refinement

*R*[*F* ^2^ > 2σ(*F* ^2^)] = 0.028
*wR*(*F* ^2^) = 0.082
*S* = 1.04
4050 reflections
152 parameters
H atoms treated by a mixture of independent and constrained refinement
Δρ_max_ = 0.49 e Å^−3^
Δρ_min_ = −0.41 e Å^−3^

### 

Data collection: *APEX2* (Bruker, 2009 ▶); cell refinement: *SAINT* (Bruker, 2009 ▶); data reduction: *SAINT*; program(s) used to solve structure: *SHELXTL* (Sheldrick, 2008 ▶); program(s) used to refine structure: *SHELXTL*; molecular graphics: *SHELXTL*; software used to prepare material for publication: *SHELXTL* and *PLATON* (Spek, 2009 ▶).

## Supplementary Material

Crystal structure: contains datablocks global, I. DOI: 10.1107/S1600536811004107/lh5199sup1.cif

Structure factors: contains datablocks I. DOI: 10.1107/S1600536811004107/lh5199Isup2.hkl

Additional supplementary materials:  crystallographic information; 3D view; checkCIF report

## Figures and Tables

**Table 1:** Hydrogen-bond geometry (Å, °)

*D*—H⋯*A*	*D*—H	H⋯*A*	*D*⋯*A*	*D*—H⋯*A*
N2—H1*N*2⋯O3^i^	0.862 (15)	2.009 (14)	2.8553 (10)	167.0 (13)
O1*W*—H1*W*1⋯O2^ii^	0.89 (2)	1.95 (2)	2.8289 (9)	171.5 (19)
N2—H2*N*2⋯O1*W*	0.875 (16)	2.055 (16)	2.9139 (11)	166.9 (15)
O1*W*—H2*W*1⋯O2^iii^	0.822 (19)	2.007 (19)	2.8270 (10)	175 (2)
C1—H1*A*⋯O1^iv^	0.93	2.57	3.4076 (11)	150
C2—H2*A*⋯O1*W*^v^	0.93	2.58	3.3595 (11)	142
C6—H6*A*⋯O3^i^	0.97	2.53	3.3160 (11)	138
C6—H6*B*⋯O1^iv^	0.97	2.52	3.2589 (11)	133

Symmetry codes: (i) 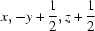; (ii) 
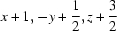
; (iii) 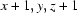; (iv) 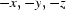; (v) 
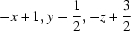
.

**Fig. 1.**
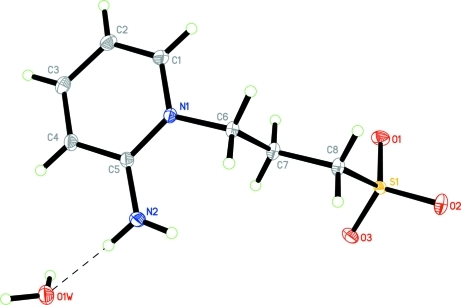
The molecular structure, showing 50% probability displacement ellipsoids. Hydrogen atoms are shown as spheres of arbitrary radius. The dashed line indicates a hydrogen bond.

**Fig. 2.**
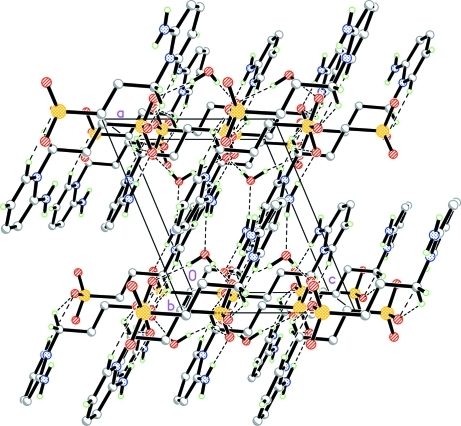
The crystal packing of (I) viewed along the b axis. Dashed lines indicate hydrogen bonds. H atoms not involved in the hydrogen bond interactions have been omitted for clarity.

## supplementary crystallographic information

### Comment

The sulfopropyl group has been widely used as a hydrophilic enhancing agent in dye, nucleocides, proteins and polymers (Adamczyk & Rege, 1998). In addition, derivatives of sulfopropylated compounds are used extensively in both manufacturing and diagnostic industries. For example, sulfopropylated fatty acids have been found to possess antistatic properties while sulfopropylated acridines have been used industrially as chemiluminescent probes (Adamczyk & Rege, 1998). These properties of sultone can be acredited to the CH_2_ group attached to the S atom which allowed attachment to other organic fragments such as the 2-amino pyridine group in the current study. The indisputable application of 2-aminopyridine in the synthesis of pharmaceuticals such as antihistamines and piroxican has been the main reason for its substantial desirability up to now (Salimon *et al.*, 2009). In this study, sultone was reacted with 2-aminopyridine and attachment was achieved through the N atom in the ring. This compound allows the immobilization onto silica to serve as a heterogeneous catalyst in various industrial applications (Jayamurugan *et al.*, 2009).

All parameters in the title compound (I), Fig. 1, are within normal ranges and comparable to a related structure (Koclega *et al.*, 2007). The torsion angles S1-C8-C7-C6 and N1-C6-C7-C8 are -178.36 (5) and -179.58 (6)° respectively. In the selected asymmetric unit, the 2-amino-N-3-sulfatepropyl-pyridinium molecule is linked to the water molecule through an N2—H2N2···O1W (Table 1, Fig. 1) intermolecular hydrogen bond.

In the crystal structure, intermolecular O1W—H1W1···O2^ii^, O1W—H2W1···O2^iii^, N2—H1N2···O3^i^, C6—H6B···O1^iv^, C1—H1A···O1^iv^, C2—H2A···O1W^v^ and weak C6—H6A···O3^i^ hydrogen bnods (Table 1, Fig. 2) link molecules into a three-dimensional network. The weak C6—H6B···O1^iv^ interactions are involved in R_2_^2^(12) ring motifs while weak C1—H1A···O1^iv^ interactions form R_1_^2^ (6) ring motifs (Bernstein *et al.*, 1995).

### Experimental

2-amino pyridine (3g, 1.9 mmol) was dissolved in acetonitrile (20 ml). 1,3-propane sultone (2.8 ml, 1.9 mmol)  was added to the mixture and was refluxed at 353 K for 1 h. The light yellowish precipitate was filtered and washed with acetonitrile (10 ml) and diethyl ether (10 ml). The product was recrystallized in methanol: water (9:1 ratio) to produce light yellow needle-shaped crystals.

### Refinement

H atoms attached to N and O atoms were located from difference Fourier map and freely refined. The remaining H atoms were positioned geometrically [C-H = 0.93 and 0.97Å] and refined using a riding model, with U_iso_(H) = 1.2U_eq_(C).

### Crystal data

**Table:**

C_8_H_12_N_2_O_3_S·H_2_O	*F*(000) = 496
*M**_r_* = 234.27	*D*_x_ = 1.503 Mg m^−^^3^
Monoclinic, *P*2_1_/*c*	Mo *K*α radiation, λ = 0.71073 Å
Hall symbol: -P 2ybc	Cell parameters from 7571 reflections
*a* = 9.0771 (3) Å	θ = 3.2–33.6°
*b* = 16.6307 (7) Å	µ = 0.31 mm^−^^1^
*c* = 7.4393 (3) Å	*T* = 100 K
β = 112.794 (1)°	Needle, light-yellow
*V* = 1035.32 (7) Å^3^	0.51 × 0.14 × 0.14 mm
*Z* = 4	

### Data collection

**Table:**

Bruker SMART APEXII CCD area-detector diffractometer	4050 independent reflections
Radiation source: fine-focus sealed tube	3694 reflections with *I* > 2σ(*I*)
graphite	*R*_int_ = 0.023
φ and ω scans	θ_max_ = 33.6°, θ_min_ = 3.2°
Absorption correction: multi-scan (*SADABS*; Bruker, 2009)	*h* = −14→13
*T*_min_ = 0.858, *T*_max_ = 0.958	*k* = −24→25
15075 measured reflections	*l* = −11→11

### Refinement

**Table:**

Refinement on *F*^2^	Primary atom site location: structure-invariant direct methods
Least-squares matrix: full	Secondary atom site location: difference Fourier map
*R*[*F*^2^ > 2σ(*F*^2^)] = 0.028	Hydrogen site location: inferred from neighbouring sites
*wR*(*F*^2^) = 0.082	H atoms treated by a mixture of independent and constrained refinement
*S* = 1.04	*w* = 1/[σ^2^(*F*_o_^2^) + (0.0443*P*)^2^ + 0.3224*P*] where *P* = (*F*_o_^2^ + 2*F*_c_^2^)/3
4050 reflections	(Δ/σ)_max_ < 0.001
152 parameters	Δρ_max_ = 0.49 e Å^−^^3^
0 restraints	Δρ_min_ = −0.41 e Å^−^^3^

### Special details

**Table:**

Experimental. The crystal was placed in the cold stream of an Oxford Cyrosystems Cobra open-flow nitrogen cryostat (Cosier & Glazer, 1986) operating at 100.0 (1) K.
Geometry. All esds (except the esd in the dihedral angle between two l.s. planes) are estimated using the full covariance matrix. The cell esds are taken into account individually in the estimation of esds in distances, angles and torsion angles; correlations between esds in cell parameters are only used when they are defined by crystal symmetry. An approximate (isotropic) treatment of cell esds is used for estimating esds involving l.s. planes.
Refinement. Refinement of F^2^ against ALL reflections. The weighted R-factor wR and goodness of fit S are based on F^2^, conventional R-factors R are based on F, with F set to zero for negative F^2^. The threshold expression of F^2^ > 2sigma(F^2^) is used only for calculating R-factors(gt) etc. and is not relevant to the choice of reflections for refinement. R-factors based on F^2^ are statistically about twice as large as those based on F, and R- factors based on ALL data will be even larger.

### Fractional atomic coordinates and isotropic or equivalent isotropic displacement parameters (Å^2^)

**Table:**

	*x*	*y*	*z*	*U*_iso_*/*U*_eq_	
S1	0.02015 (2)	0.135731 (11)	−0.20477 (3)	0.00995 (6)	
O1	0.04125 (8)	0.05139 (4)	−0.23882 (9)	0.01617 (12)	
O2	−0.13259 (7)	0.16825 (4)	−0.34346 (9)	0.01625 (12)	
O3	0.15490 (7)	0.18591 (4)	−0.19553 (9)	0.01467 (12)	
N1	0.29524 (8)	0.09165 (4)	0.54330 (9)	0.01030 (12)	
N2	0.40686 (9)	0.22107 (4)	0.57767 (11)	0.01460 (13)	
C1	0.30927 (10)	0.01161 (5)	0.59030 (12)	0.01312 (14)	
H1A	0.2253	−0.0229	0.5236	0.016*	
C2	0.44337 (10)	−0.01854 (5)	0.73247 (12)	0.01495 (14)	
H2A	0.4531	−0.0733	0.7604	0.018*	
C3	0.56716 (10)	0.03537 (5)	0.83638 (12)	0.01397 (14)	
H3A	0.6577	0.0167	0.9384	0.017*	
C4	0.55426 (9)	0.11494 (5)	0.78738 (11)	0.01314 (14)	
H4A	0.6364	0.1502	0.8558	0.016*	
C5	0.41636 (9)	0.14415 (5)	0.63289 (11)	0.01089 (13)	
C6	0.14495 (9)	0.11861 (5)	0.38687 (11)	0.01081 (13)	
H6A	0.1216	0.1734	0.4122	0.013*	
H6B	0.0575	0.0846	0.3847	0.013*	
C7	0.16001 (9)	0.11457 (5)	0.19015 (11)	0.01233 (13)	
H7A	0.2483	0.1482	0.1932	0.015*	
H7B	0.1829	0.0597	0.1649	0.015*	
C8	0.00708 (9)	0.14264 (5)	0.02759 (11)	0.01198 (13)	
H8A	−0.0140	0.1980	0.0514	0.014*	
H8B	−0.0815	0.1100	0.0275	0.014*	
O1W	0.71613 (8)	0.30058 (4)	0.75218 (10)	0.01777 (13)	
H1N2	0.3233 (17)	0.2419 (8)	0.489 (2)	0.020 (3)*	
H1W1	0.772 (2)	0.3125 (10)	0.877 (3)	0.034 (4)*	
H2N2	0.4915 (18)	0.2508 (9)	0.636 (2)	0.025 (3)*	
H2W1	0.765 (2)	0.2638 (11)	0.726 (3)	0.041 (5)*	

### Atomic displacement parameters (Å^2^)

**Table:**

	*U*^11^	*U*^22^	*U*^33^	*U*^12^	*U*^13^	*U*^23^
S1	0.01095 (9)	0.01021 (9)	0.00793 (9)	−0.00137 (6)	0.00282 (6)	−0.00137 (5)
O1	0.0228 (3)	0.0105 (3)	0.0159 (3)	−0.0020 (2)	0.0082 (2)	−0.0038 (2)
O2	0.0142 (3)	0.0222 (3)	0.0094 (2)	0.0024 (2)	0.0014 (2)	0.0011 (2)
O3	0.0156 (3)	0.0152 (3)	0.0138 (3)	−0.0060 (2)	0.0064 (2)	−0.0030 (2)
N1	0.0103 (3)	0.0104 (3)	0.0087 (3)	0.0005 (2)	0.0021 (2)	0.0004 (2)
N2	0.0123 (3)	0.0107 (3)	0.0165 (3)	−0.0005 (2)	0.0008 (2)	0.0015 (2)
C1	0.0153 (3)	0.0109 (3)	0.0126 (3)	−0.0007 (2)	0.0048 (3)	0.0004 (2)
C2	0.0169 (3)	0.0123 (3)	0.0147 (3)	0.0022 (3)	0.0051 (3)	0.0029 (3)
C3	0.0133 (3)	0.0157 (3)	0.0121 (3)	0.0033 (3)	0.0041 (3)	0.0022 (3)
C4	0.0110 (3)	0.0146 (3)	0.0115 (3)	0.0007 (3)	0.0018 (2)	0.0001 (3)
C5	0.0103 (3)	0.0112 (3)	0.0102 (3)	0.0003 (2)	0.0029 (2)	−0.0005 (2)
C6	0.0097 (3)	0.0128 (3)	0.0087 (3)	0.0008 (2)	0.0023 (2)	0.0003 (2)
C7	0.0113 (3)	0.0160 (3)	0.0090 (3)	0.0020 (3)	0.0032 (2)	0.0003 (2)
C8	0.0108 (3)	0.0156 (3)	0.0085 (3)	0.0015 (2)	0.0027 (2)	−0.0001 (2)
O1W	0.0167 (3)	0.0174 (3)	0.0147 (3)	0.0013 (2)	0.0010 (2)	−0.0018 (2)

### Geometric parameters (Å, °)

**Table:**

S1—O1	1.4511 (6)	C3—C4	1.3655 (12)
S1—O3	1.4602 (6)	C3—H3A	0.9300
S1—O2	1.4734 (6)	C4—C5	1.4174 (11)
S1—C8	1.7817 (8)	C4—H4A	0.9300
N1—C5	1.3593 (10)	C6—C7	1.5231 (11)
N1—C1	1.3695 (10)	C6—H6A	0.9700
N1—C6	1.4786 (10)	C6—H6B	0.9700
N2—C5	1.3361 (10)	C7—C8	1.5188 (11)
N2—H1N2	0.861 (14)	C7—H7A	0.9700
N2—H2N2	0.874 (15)	C7—H7B	0.9700
C1—C2	1.3616 (11)	C8—H8A	0.9700
C1—H1A	0.9300	C8—H8B	0.9700
C2—C3	1.4110 (12)	O1W—H1W1	0.892 (17)
C2—H2A	0.9300	O1W—H2W1	0.825 (19)
			
O1—S1—O3	113.32 (4)	C5—C4—H4A	119.8
O1—S1—O2	112.62 (4)	N2—C5—N1	121.38 (7)
O3—S1—O2	111.53 (4)	N2—C5—C4	120.60 (7)
O1—S1—C8	107.14 (4)	N1—C5—C4	118.02 (7)
O3—S1—C8	106.67 (4)	N1—C6—C7	110.12 (6)
O2—S1—C8	104.92 (4)	N1—C6—H6A	109.6
C5—N1—C1	121.44 (7)	C7—C6—H6A	109.6
C5—N1—C6	121.03 (7)	N1—C6—H6B	109.6
C1—N1—C6	117.50 (6)	C7—C6—H6B	109.6
C5—N2—H1N2	123.5 (9)	H6A—C6—H6B	108.2
C5—N2—H2N2	116.6 (10)	C8—C7—C6	110.91 (6)
H1N2—N2—H2N2	119.9 (14)	C8—C7—H7A	109.5
C2—C1—N1	121.53 (7)	C6—C7—H7A	109.5
C2—C1—H1A	119.2	C8—C7—H7B	109.5
N1—C1—H1A	119.2	C6—C7—H7B	109.5
C1—C2—C3	118.30 (8)	H7A—C7—H7B	108.0
C1—C2—H2A	120.9	C7—C8—S1	111.64 (6)
C3—C2—H2A	120.9	C7—C8—H8A	109.3
C4—C3—C2	120.12 (7)	S1—C8—H8A	109.3
C4—C3—H3A	119.9	C7—C8—H8B	109.3
C2—C3—H3A	119.9	S1—C8—H8B	109.3
C3—C4—C5	120.41 (8)	H8A—C8—H8B	108.0
C3—C4—H4A	119.8	H1W1—O1W—H2W1	105.9 (16)
			
C5—N1—C1—C2	1.82 (12)	C3—C4—C5—N2	−176.43 (8)
C6—N1—C1—C2	−179.98 (7)	C3—C4—C5—N1	3.44 (12)
N1—C1—C2—C3	2.11 (12)	C5—N1—C6—C7	87.55 (9)
C1—C2—C3—C4	−3.15 (12)	C1—N1—C6—C7	−90.66 (8)
C2—C3—C4—C5	0.38 (13)	N1—C6—C7—C8	−179.58 (6)
C1—N1—C5—N2	175.31 (8)	C6—C7—C8—S1	−178.36 (5)
C6—N1—C5—N2	−2.83 (12)	O1—S1—C8—C7	62.61 (7)
C1—N1—C5—C4	−4.56 (11)	O3—S1—C8—C7	−59.04 (7)
C6—N1—C5—C4	177.31 (7)	O2—S1—C8—C7	−177.48 (6)

### Hydrogen-bond geometry (Å, °)

**Table:**

*D*—H···*A*	*D*—H	H···*A*	*D*···*A*	*D*—H···*A*
N2—H1N2···O3^i^	0.862 (15)	2.009 (14)	2.8553 (10)	167.0 (13)
O1W—H1W1···O2^ii^	0.89 (2)	1.95 (2)	2.8289 (9)	171.5 (19)
N2—H2N2···O1W	0.875 (16)	2.055 (16)	2.9139 (11)	166.9 (15)
O1W—H2W1···O2^iii^	0.822 (19)	2.007 (19)	2.8270 (10)	175 (2)
C1—H1A···O1^iv^	0.93	2.57	3.4076 (11)	150
C2—H2A···O1W^v^	0.93	2.58	3.3595 (11)	142
C6—H6A···O3^i^	0.97	2.53	3.3160 (11)	138
C6—H6B···O1^iv^	0.97	2.52	3.2589 (11)	133

Symmetry codes: (i) *x*, −*y*+1/2, *z*+1/2; (ii) *x*+1, −*y*+1/2, *z*+3/2; (iii) *x*+1, *y*, *z*+1; (iv) −*x*, −*y*, −*z*; (v) −*x*+1, *y*−1/2, −*z*+3/2.
